# Three-Dimensional Geometric Analysis of Felid Limb Bone Allometry

**DOI:** 10.1371/journal.pone.0004742

**Published:** 2009-03-09

**Authors:** Michael Doube, Alexis Wiktorowicz Conroy, Per Christiansen, John R. Hutchinson, Sandra Shefelbine

**Affiliations:** 1 Department of Bioengineering, Imperial College London, South Kensington, London, United Kingdom; 2 Structure and Motion Laboratory, The Royal Veterinary College, Hatfield, Hertfordshire, United Kingdom; 3 Zoological Museum, Universitetsparken, Copenhagen, Denmark; University of Liverpool, United Kingdom

## Abstract

**Background:**

Studies of bone allometry typically use simple measurements taken in a small number of locations per bone; often the midshaft diameter or joint surface area is compared to body mass or bone length. However, bones must fulfil multiple roles simultaneously with minimum cost to the animal while meeting the structural requirements imposed by behaviour and locomotion, and not exceeding its capacity for adaptation and repair. We use entire bone volumes from the forelimbs and hindlimbs of Felidae (cats) to investigate regional complexities in bone allometry.

**Method/Principal Findings:**

Computed tomographic (CT) images (16435 slices in 116 stacks) were made of 9 limb bones from each of 13 individuals of 9 feline species ranging in size from domestic cat (*Felis catus*) to tiger (*Panthera tigris*). Eleven geometric parameters were calculated for every CT slice and scaling exponents calculated at 5% increments along the entire length of each bone. Three-dimensional moments of inertia were calculated for each bone volume, and spherical radii were measured in the glenoid cavity, humeral head and femoral head. Allometry of the midshaft, moments of inertia and joint radii were determined. Allometry was highly variable and related to local bone function, with joint surfaces and muscle attachment sites generally showing stronger positive allometry than the midshaft.

**Conclusions/Significance:**

Examining whole bones revealed that bone allometry is strongly affected by regional variations in bone function, presumably through mechanical effects on bone modelling. Bone's phenotypic plasticity may be an advantage during rapid evolutionary divergence by allowing exploitation of the full size range that a morphotype can occupy. Felids show bone allometry rather than postural change across their size range, unlike similar-sized animals.

## Introduction

As animals become more massive, their skeletons must increase in size to accommodate increased body volume and increase in strength in order to sustain larger applied loads. Scaling studies have attempted to determine a relationship between animal size and bone length and diameter, as well as other correlates of bone strength such as cross-sectional area and bone curvature to determine comparative trends in strength-mass relationships [1]. A common approach in scaling studies is to use linear measures of bone dimensions [2]–[5]: length and midshaft diameter are both easily obtained and are predictive of bending and buckling behaviour in simple, materially homogeneous beams. However, most bones exist in complicated mechanical environments where they must articulate with other bones, provide tendon and ligament attachment sites, recesses for bulky organs, and accommodate gaits and behaviours. By failing to account for these functional complexities, simple scaling approaches have overlooked critical variations in bone shape.

Bone shapes result from embryonic development, phenotypic plasticity (modelling) and evolution. The initial position, size and shape of cartilaginous anlagen are determined directly by the genome during embryonic skeletogenesis and form a template for bone growth [6], [7]. Anlagen are replaced by endochondral ossification forming bones that are modified continuously throughout an animal's life [8]. Fetal muscle contraction is necessary for the normal development of bone size and shape, indicating that bone shape is influenced by the mechanical environment from an early developmental stage [9], [10]. Bone modelling occurs in response to (at least) strain magnitude, strain rate and the presence of overlying soft tissue [11], [12]. Bone tissue is a complex, mineralised, fibre-reinforced porous composite material that displays regional anisotropy at multiple levels of organisation [13]–[17] and which exists within a spectrum of tissue damage and repair [18]–[21]. Monotonic (traumatic) fracture may result in severe lameness or death but many animals survive with healed bones [22]–[24], while fatigue failure of bones is painful and reduces locomotor performance even in the absence of complete bone fracture [25].

Cross-sectional geometric measurements have been used to advance estimates of bone strength beyond what is possible with simple diameter measurements. Cortical thickness has been indirectly deduced from computed tomography (CT) images at the mid-shaft [26], [27], and has been measured on radiographs [28]. These studies assumed that diaphyseal bone approximates to a cylinder [26] or elliptical beam [28] and calculated bone strength for a homogeneous beam of constant cross section. Selker and Carter calculated the polar second moment of area (*J*_z_) from CT images of the bone at midshaft in artiodactyls and calculated a bone strength index (*S*_b_) from *J*_z_, midshaft diameter (*d*) and length (*l*: *S*_b_ = *J*_z_/*d l*). They found that the bone strength index scaled similarly across species despite differences in scaling of length and diameter [26].

Christiansen found that bones from larger species appeared to scale with lower regression coefficients than bones from smaller species, and eschewed the possibility that a single scaling exponent could explain all variation in bone shape, on the grounds that it would not accommodate variations in posture or locomotor style [5]. Bertram and Biewener had previously suggested that scaling exponents varied within clades of different body mass ranges and that posture changed with increasing body mass, as large animals' limbs were relatively vertical and less crouched than small animals [4]. Straightening the limbs with increasing size keeps bone bending and muscular stresses nearly constant by increasing muscle moment arms and reducing joint moments [29].

By restricting this study to a single morphotype (felids), we aim to avoid the effects of gross postural and behavioural change that can confound attempts to study size effects on bone scaling [30]. Davis selected Felidae for his organ weight and limb length scaling study, and noted that felids approached the experimental ideal of consistent morphology and behaviour across their size range [31]. Day and Jayne demonstrated that posture and gait were not correlated with body mass within 9 felid species [32]. Since posture does not change with body size in Felidae, the role of allometry in maintaining relative bone strength should be marked. But would this allometry be fully defined by midshaft parameters as has been shown previously (Table 1), or could there be hidden complexities and mechanisms that can only be found by measuring mechanically-relevant geometric parameters throughout the whole bone? Here, we image and analyse scapulae and 8 fore- and hindlimb long bones from 9 felid species in three dimensions. We determine geometrical parameters along the entire length of each bone to reveal scaling relationships localised to individual bone regions. This uses a novel method that we have developed, which can be applied to skeletons quickly and semi-automatically.

**Table 1:** Felid midshaft scaling exponents.

Study	Comparison	Bone	*A*
Bertram & Biewener (1990) *	*D* ∝ *l^a^*; *D* = midshaft craniocaudal diameter (1)	Humerus	1.38
		Radius	1.49
		Femur	1.16
		Tibia	1.40
	*D* ∝ *l^a^*; *D* = midshaft mediolateral diameter (1)	Humerus	1.23
		Radius	1.48
		Femur	1.14
		Tibia	1.35
Anyonge (1993) #	*M* ∝ *x^a^*; *x* parameters are listed		
	Length (3)	Femur	3.20
	Midshaft circumference (3)		2.92
	Midshaft cross-sectional area (1.5)		1.31
	Midshaft mediolateral second moment of area (0.75)		0.69
	Midshaft craniocaudal second moment of area (0.75)		0.71
	Distal articular area (1.5)		1.31
	Length (3)	Humerus	3.13
	Midshaft circumference (3)		2.65
	Midshaft cross-sectional area (1.5)		1.25
	Midshaft mediolateral second moment of area (0.75)		0.63
	Midshaft craniocaudal second moment of area (0.75)		0.64

Scaling exponents (*a*) from previous studies on felid skeletons [4], [28]. Scaling relationships reported by Bertram and Biewener (1990) have been inverted. Isometric scaling exponents are indicated in parentheses.

*M*, body mass; *l*, length.

Species included: * *Acinonyx jubatus*, *Felis aurata*, *F. bengalensis*, *F. catus*, *F. chaus*, *F. colocolo*, *F. concolor*, *F. geoffroyi*, *F. libyca*, *F. manul*, *F. marmorata*, *F. margarita*, *F. pardalis*, *F. pleniceps*, *F. serval*, *F. tigrina*, *F. viverrina*, *F. wiedii*, *F. yaguarundi*, *Panthera leo*, *P. onca*, *P. pardus*, *P. tigris*, *Neofelis nebulosa*, *Uncia uncia*, *Lynx caracal*, *L. lynx*, *L. rufus*; # *N. nebulosa*, *F. caracal*, *F. pardalis*, *A. jubatus*, *P. onca*, *P. pardus*, *P. leo*, *P. tigris*, *F. serval*, *F. lybica*, *F. yagouaroundi*, *L. rufus*, *Puma concolor*, *U. uncia*. Taxonomic classification is as reported by the original authors.

## Results

Phylogenetic analysis indicated that scaling patterns and correlations were not constrained by phylogenetic topology when tested on 50% length (midshaft) data (Table S1), meaning that phylogeny could be defensibly ignored in further scaling calculations. Scaling exponents at the midshaft were generally greater than isometry, except cross-sectional area of the fibula and cortical thickness, which scaled less than isometry. Allometry of cross-sectional area, second moment of area and diameter versus length were within the ranges described by previous authors (Table 1), either through comparison with similar measurements or exponents calculated using *M* as an intermediate [4], [28]. Scapular diameter scaled to length with an exponent of 4.08±2.13, much greater than isometry (Table 2). All further scaling relationships were calculated with no set intercept, an assumption of phylogenetic independence and without averaging measurements from multiple animals of single species prior to regression calculation.

**Table 2:** Midslice scaling exponents.

Bone	*a*±*CI* for variables (*y*) where *y* ∝ *l^a^*					
	*d*_max_ (1)	*t*_av_ (1)	*CSA* (2)	*Z*_max_ (3)	*I*_max_ (4)	*J*_z_ (4)
Scapula	**4.08±2.13**	n.c.	2.28±0.53	3.47±0.75	4.50±0.80	4.35±0.70
Humerus	1.36±0.46	0.62±0.51	2.13±0.58	3.53±0.97	4.92±1.45	4.54±1.27
Radius	1.34±0.47	0.85±0.36	2.48±0.81	3.97±1.23	5.34±1.73	5.34±1.68
Ulna	1.24±0.55	0.82±0.50	2.37±1.02	3.66±1.46	4.88±2.04	5.02±2.06
Third Metacarpal	1.06±0.21	**0.58±0.22**	1.90±0.42	3.14±0.65	4.22±0.85	4.20±0.78
Femur	1.11±0.27	0.89±0.24	2.10±0.46	3.25±0.72	4.38±0.97	4.40±0.99
Tibia	1.19±0.21	1.02±0.27	2.34±0.47	3.55±0.66	4.78±0.85	4.83±0.88
Fibula	1.24±0.75	n.c.	1.44±0.77	2.79±1.65	4.15±2.46	3.97±2.26
Third Metatarsal	1.26±0.35	**0.61±0.27**	2.13±0.58	3.56±0.85	4.85±1.13	4.91±1.17

Phylogenetically corrected scaling exponents, *a*±95% CI, were calculated for midshaft parameters (*y*) versus bone length (*l*). The exponent expected for the isometric case is in parentheses. Statistically significant (p<0.05) relationships are indicated in bold. Scaling exponents tend to be greater than isometry, except for cortical thickness, which appears to scale at less than isometry. Full tables including correlation coefficients (*r*) and standard error (SE) are provided as supplementary information (Table S1).

*d*_max_, maximum external diameter.

*t*_av_, mean cortical thickness.

*CSA*, cross-sectional area.

*Z*_max_, maximum section modulus.

*I*_max_, maximum second moment of area.

*J*_z_, polar moment of inertia.

n.c., not calculated.

**Figure 1:**
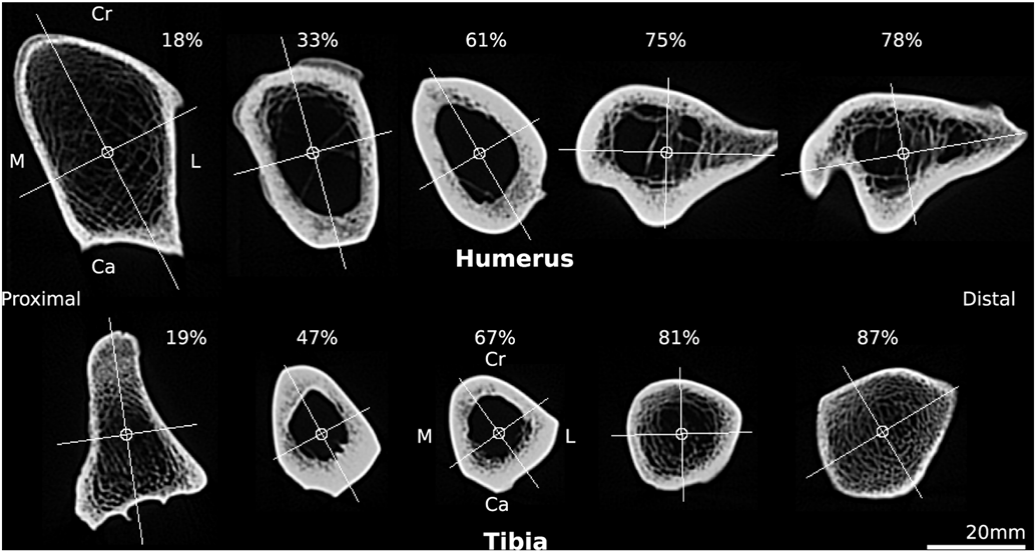
Computed tomograms of a tiger humerus and tibia. Selected CT slices from the humerus and tibia of *Panthera tigris* (tiger) with centroids and principal axes shown. Note the substantial variation in cortical shape and thickness along the length of each bone. Per cent length is indicated for each slice; 0% is most proximal. Cr, cranial; Ca, caudal; M, medial; L, lateral.

Visual inspection of bone cross-sections indicated that assumptions of circularity or elliptical geometry underestimate the complexity of bone structure (Figure 1), which diverged substantially between and within bones.

**Figure 2:**
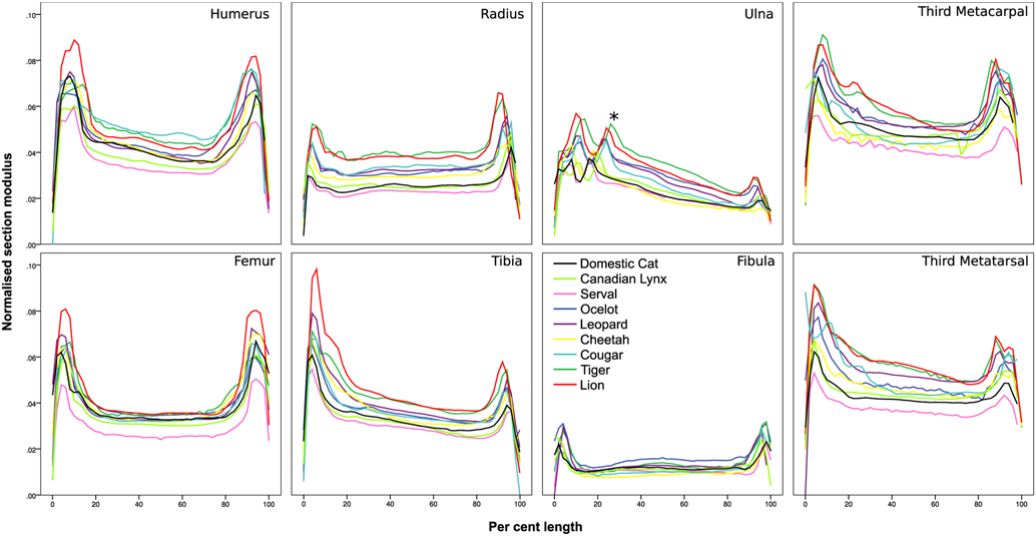
Normalised section modulus versus per cent length. Normalised section modulus (*Z*_max_^1/3^ / length) versus per cent length (proximal = 0%) for appendicular long bones. Contributions from multiple individuals within species have been averaged. Note the characteristic profile of each bone, a tendency for epiphyses to have markedly larger normalised section moduli than diaphyses and that larger felids tend to have larger normalised section moduli at all per cent lengths than smaller felids in all bones except the fibula. Coronoid process (*). See Figure 3E for reference bones.

Normalised section modulus (*Z*_max_^1/3^ / *l*) versus per cent length plots (Figure 2) showed characteristic profiles for each bone, with larger felids generally having greater normalised section modulus at each per cent length than smaller felids. In addition, epiphyses had noticeably greater normalised section moduli than diaphyses. Normalised section moduli tended to be greater proximally than distally, which was most pronounced in the ulna, tibia and larger felids' metapodials. The coronoid process and trochlear notch of the ulna appear to become displaced distally in larger felids (Figure 2).

**Figure 3:**
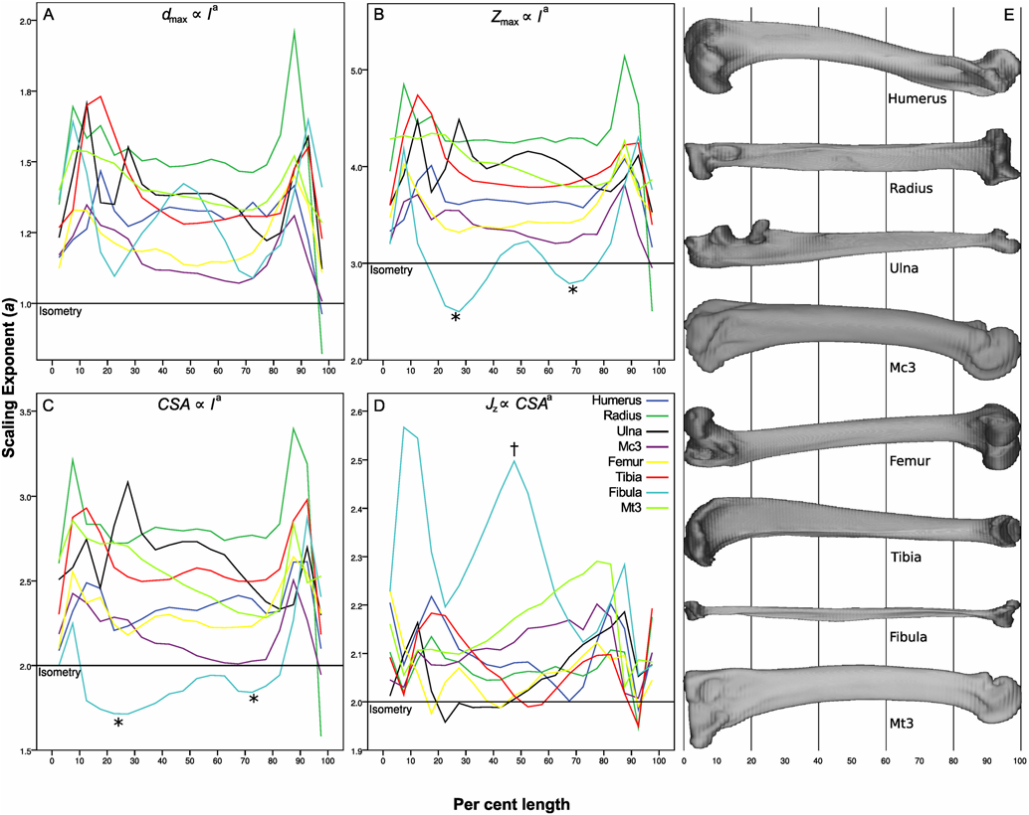
Scaling exponent (*a*) versus per cent length. Scaling exponents (*a*) were calculated for 5% length bins for each bone (0% is most proximal) for the following allometric relationships: (A) *d*_max_ ∝ *l* ^a^; (B) *Z*_max_ ∝ *l* ^a^; (C) *CSA* ∝ *l* ^a^; (D) *J*_z_ ∝ *CSA* ^a^. The isometric exponent is indicated by a horizontal line in each plot. Wide variation is evident in scaling exponents both between and within bones. Scaling exponents tend to be greater at the epiphyses that at 50% length, and in the fibula the proximal and distal thirds (*) scale less strongly than the midshaft (†). (E) *Panthera pardus* (leopard) bones scaled to 100% length for reference.

Plots of scaling exponent versus per cent length showed wide variation in scaling exponents between and within bones (Figure 3). The radius, ulna and tibia were more strongly allometric than the humerus and femur. There was a general trend towards greater scaling exponents at the epiphyses than at the diaphyses. Polar moment of area scaled to cross-sectional area with exponents generally greater than isometry, indicating placement of bone relatively more distant from the centroid as cross-sectional area increases (Figure 3D). This would result in a larger diameter and thinner cortex than would be expected with isometry, resulting in a mechanical strength increase over bones of similar mass but with relatively thicker cortices and smaller outer diameters. Comparison of scaling exponent versus per cent length plots with whole-bone anatomy indicated relationships between scaling and anatomical features; for example the maximum diameter of the tibia scales strongly positive in the region of the tibial crest (10% length), as does the midshaft of the fibula in the region of M. peroneus brevis' origin (40–60% length). Diameter (Figure 3A), section modulus (Figure 3B) and cross-sectional area (Figure 3C) versus per cent length scaling of the ulna also varies within bone length, showing stronger positive allometry at 50% than at 85% length, and displaying increases in allometric exponent that relate to the positions of the anconeal and coronoid processes.

Allometry of the glenoid cavity, femoral head and humeral head radii versus bone length was present, with the glenoid cavity showing allometry consistent with the static stress similarity hypothesis (*D* ∝ *l* ^3/2^) (Table 3). Scaling exponents for humeral (*a* = 1.34) and femoral (*a* = 1.26) head radii versus length were greater than 1, indicating that the shoulder and hip joint surface areas increase allometrically with increasing body size. This compared well with previous allometric calculations of the distal articular area (*A*) of the felid femur, which showed *M* ∝ *A* ^1.31^ (Table 1), and because *M* ∝ *l* ^3.20^, *A* ∝ *l* ^2.44^ and distal articular ‘radius’ ∝ *l* ^1.22^.

**Table 3:** Glenoid cavity, femoral head and humeral head allometry.

Comparison	*a*	95% CI
Femoral head radius ∝ femoral length*^a^*	1.26*	1.10–1.46
Femoral head radius ∝ femoral midshaft diameter*^a^*	1.12	1.00–1.24
Humeral head radius ∝ humeral length*^a^*	1.34*	1.17–1.54
Humeral head radius ∝ femoral head radius*^a^*	1.08	0.99–1.18
Humeral head radius ∝ humeral midshaft diameter*^a^*	1.01	0.90–1.12
Glenoid radius ∝ humeral head radius*^a^*	1.15	1.03–1.28
Glenoid radius ∝ scapular length*^a^*	1.48*	1.28–1.71

Scaling exponents (*a*) and 95% confidence intervals (CI) for femoral head, humeral head and glenoid cavity spherical radii against bone lengths and diameters. The strongest allometry is evident between articular radii and bone lengths (*), which are strongly related to body size.

Isometry: *a* = 1.0.

**Figure 4:**
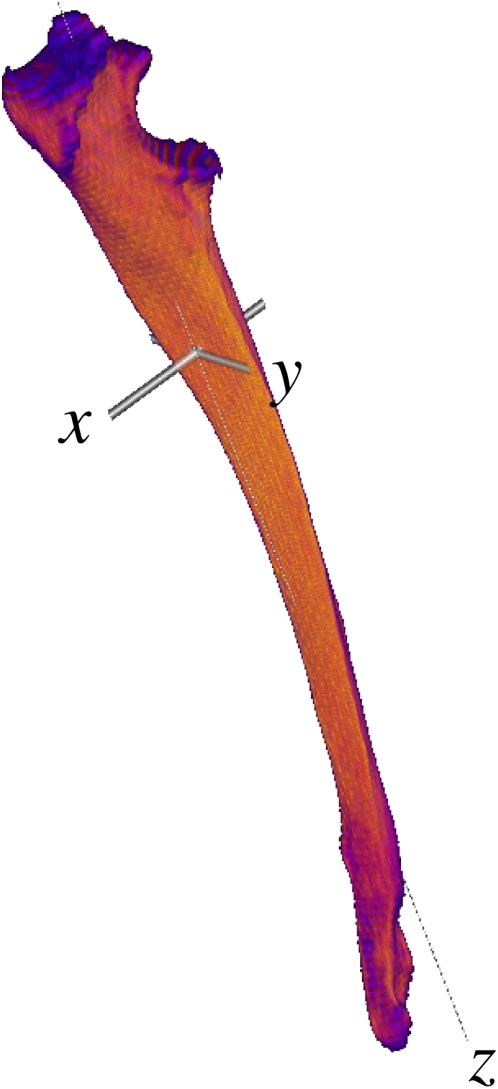
Principal axes. *Acinonyx jubatus* (cheetah) ulna with principal axes intersecting at the 3D centroid. Note the proximal displacement of the centroid relative to 50% length. *I*_1_ and *I*_2_ are the moments of inertia around the *x* and *y* axes; *I*_3_ is the moment of inertia around *z*.

The minimum axis of inertia generally corresponded very closely to the direction of the *z*-scan axis, meaning that CT slices were good representations of axial cross-sectional geometry (Figure 4). The two greatest moments of inertia (*I*_1_ and *I*_2_) were not significantly different from each other (p = 0.93), so scaling exponents were calculated only for the least moment of inertia (*I*_3_) versus the greatest moment of inertia (*I*_1_: Table 4). Scaling exponents for moments of inertia ranged from isometric (scapula, radius, third metacarpal) to mild allometry (humerus, ulna, femur, tibia, fibula, third metatarsal). Scaling exponents >1 indicate placement of bone relatively more distant from the long axis as bones become longer, which is consistent with other findings such as positive allometry of section modulus despite the trend towards negative allometry of cortical thickness (Table 2; Figure 3).

**Table 4:** Moments of inertia allometry.

Bone	*a* (*I*_3_ ∝ *I*_1_^a^)	95% CI
Scapula	0.99	0.96–1.01
Humerus	1.12†	1.05–1.21
Radius	1.09	0.90–1.33
Ulna	1.19†	1.11–1.28
Metacarpal	1.08	0.94–1.23
Femur	1.11†	1.06–1.17
Tibia	1.15†	1.06–1.24
Fibula	1.19†	1.02–1.38
Metatarsal	1.15†	1.04–1.26

Scaling exponents (*a*) and 95% confidence intervals (CI) for moments of inertia.

† Significantly different from isometry.

Isometry: *a* = 1.0.

## Discussion

The proposal that a general rule of scaling might apply to all skeletal structures has been gradually worn down since it was originally devised by Galileo Galilei in 1638 [1]. McMahon suggested that bones should scale with elastic similarity [33] and found some supporting evidence [3], but this was soon after discovered to be due to an “unfortunate” selection of Artiodactyla when the elastic similarity hypothesis did not generalise to other taxa [34]. Economos showed small mammals scaling more closely to isometry (*M* ∝ *l* ^3^) than large mammals which scaled more elastically (*M* ∝ *l* ^4^) [35], while Christiansen rejected both theoretical models [5]. Scaling theory has continued to evolve, to the point that bone scaling exponents are no longer assumed to fit a single model as many more factors than geometry and body mass are involved, including phylogenetic biases, joint contact pressure and muscle force allometry [1].

We have shown wide variation in scaling exponents both between and within bones from extant felids. Exploration of midshaft allometry showed exponents differing from isometry to greater or lesser extent in each bone, presumably related to individual bone functions. For example, the midshaft second moments of area of the humerus, radius and ulna scale more strongly than those of the femur, tibia and fibula, perhaps due to greater loading during intense athletic activities (including prey grasping, running, jumping, etc.) in the forelimb than the hindlimb [36]. The third metatarsal is notably longer and scales more positively than the third metacarpal bone in felids, possibly due to the functions of the forelimb and hindlimb leading to different loading environments. The major zeugopodial bones (radius, ulna, tibia) and the third metatarsal tended to show stronger allometry than the stylopodial bones (humerus, femur) and the third metacarpal. Allometry was not constant along the length of bones, generally being greater at the condyles. This suggests that scaling is highly sensitive to regional variation in bone function. In those regions where the stress on bone is expected to be mixed axial compression, bending and torsion (e.g. tibial midshaft) [19], [37]–[41] we find weak allometry, perhaps related to effective use of material to support these loading modes. It must be noted that reports of in-vivo felid-specific bone stresses or strains are scarce. Strong allometry at the epiphyses may relate to provision of articular surface area, to shear and torsion from joint loading, and tension from muscle and ligament attachment sites. Bone scaling studies may have overemphasised midshaft strength when joint stress may be just as constraining.

Crests at muscle attachment sites (humeral crest; tibial crest; greater trochanter of the femur) and hollows that accommodate muscle bellies relate directly to the presence of muscle mass while influencing the geometry and mechanics of the underlying bone. The tibial crest supplies an insertion point for the patellar ligament through which the quadriceps muscle group acts to extend the femorotibial joint. Strong allometry of cross-sectional geometry at the level of the tibial crest indicates a disproportionate increase in the femorotibial joint's extensor moment arm and effective mechanical advantage [42], perhaps enabling felids to maintain their crouched posture throughout their size range. Our data also suggest that scaling of effective mechanical advantage may occur in felid elbows due to the relative distal drift of the ulna's trochlear notch, which increases the olecranon lever arm with increasing animal size.

The tibial crest lies medial to M. tibialis cranialis, whose contractions are partly responsible for the triangular cross-section of the tibia [43]. The results of a recent simulation [44] suggested that tibial cross-sectional shape was determined largely by local periosteal surface loads (muscle contraction) whereas cross-sectional area and second moment of area were more strongly affected by the magnitude of bending and torsion (locomotor loading). Estimating regional strain and bone's mechanobiological response may help explain the varied scaling exponents within and between bones that we have observed in this study.

Since bone has a lower critical damage strain threshold in tension than compression [45], it follows that a relatively greater amount of bone must be placed in regions of tension than compression to maintain maximum strain below levels at which damage accumulates. This might explain the larger scaling exponents seen in major tendon origins and insertions. The osteogenic effect of tension is well known and regularly exploited in orthodontic and orthopaedic practice [46], [47], so allometry in tensile regions of bones may relate to muscle cross-sectional allometry and force generation. Felid muscle mass scaling was calculated by Davis to show slight positive allometry, however, the very small sample size (5 individuals, 3 species) makes this result difficult to interpret with confidence [31].

Godfrey found an isometric relationship between carnivore humeral and femoral head surface areas and their respective bone lengths [30], whereas our data demonstrate positive allometry between articular radii and bone lengths in agreement with Anyonge [28]. In the static isometric model, contact pressure increases linearly with increasing length, as *M* ∝ *l* ^3^ while *A* ∝ *l* ^2^, meaning that pressure, which is proportional to *M* / *A*, is proportional to length (i.e. *P* ∝ *l* ^3^ / *l* ^2^). Our data reveal allometric increases of joint contact areas in the shoulder and hip that forestall the pressure increase that isometric scaling would incur.

The fibula is a unique case as it transmits at most a minority of the axial load in the crus (6.4–17% in humans [48], [49]), and it correspondingly shows negative allometry in cross-sectional area and section modulus versus length. However, polar moment of area versus cross sectional area and maximum diameter versus length show marked positive allometry at around 40% length, possibly through the influence of tension from the origin of M. peroneus brevis or through being flattened against the tibia and flexor and extensor muscles of the leg.

Three-dimensional methods are useful for bones such as the scapula in which ‘midshaft’ is not applicable. Traditional mid-length scaling at the scapula gives an exponent of over 4 for diameter versus length, while moments of inertia show that the felid scapula scales isometrically, which is much more consistent with the gross appearance of felid scapulae. Broad entheses would result in less stress concentration than focussed entheses such as the triceps insertion on the olecranon, so positive allometry may not be as necessary in the scapula as it is in the epiphyses of long bones. Moment of inertia scaling (*I*_3_ ∝ *I*_1_^a^) related well to *D* ∝ *l* ^a^ scaling for the femur and tibia, indicating that moment of inertia scaling is a general approach that handles both tubular and flat bones.

Cross-sectional measurements were limited in this study to the planes perpendicular to the CT scanner's *z*-axis, which may not be the mechanical axis at each transverse level. *I*_max_ may appear artefactually increased if the mechanical axis is oblique to the CT slice, since the obliquity would cause an increase in both cross-sectional area and average distance from the centroid. Moments of inertia calculations showed that the unit vector of the bones was usually very close to alignment with the *z*-axis. Resolution was an order of magnitude less in the *z* axis than the *x–y* plane. We surmised that adjusting the plane of section was unnecessary and risked introducing interpolation errors.

Section modulus is related to tibial fatigue (‘stress’) fractures in human athletes, with those athletes with greater tibial section moduli experiencing fewer painful episodes [50]. An 87% increase in maximum second moment of area occurred in response to experimental loading of rat ulnae, which was related to 100-fold greater fatigue resistance (cycles to failure) [51], indicating that cross-sectional geometric parameters have mechanical significance for more than simply monotonic, catastrophic fracture. The relative contributions of fatigue and monotonic failure to bone evolution are unknown, and may be more or less important depending on longevity, size and behaviour. As Biewener proposed, the lifetime loading history is of central importance to this problem [42], along with the likelihood of traumatic fracture, fracture healing capacity, rate of fatigue accrual and repair and the animal's ability to cope with lameness of several weeks' duration.

Bone is a phenotypically plastic tissue; it is capable of massive changes in size and shape in response to a multitude of influences within days to weeks [8], [52], [53]. Limitations to bone's phenotypic plasticity, along with ecological and behavioural parameters, may participate in the determination of the maximum and minimum size of a given morphotype. The normal body mass range of extant felid species is approximately 1 kg–300 kg (*Prionailuris rubiginosus* - *Panthera tigris altaica*), whereas Bovidae span 2 kg–1200 kg (*Neotragus pygmaeus* – *Bubalus bubalis*). When morphotypes diverge they may be variably successful at extending into larger and smaller sizes, within the bounds of phenotypic plasticity. Most lineages examined to date use increasingly straightened limbs to maintain bone stresses within safe limits at body masses <300 kg, but use bone allometry at body masses >300 kg [42]. Felids are generally <300 kg body mass yet they do not show limb straightening; rather bone allometry and possibly reduced relative limb loading and locomotor performance are present [42]. Among mammals, felids have unusual musculoskeletal scaling upon which we have cast new light.

Genes are the units of inheritance, but they do not directly encode bone shape beyond patterning of the embryo [54]. The success of genes relates to the success of the organism that carries them; genes that regulate bone function might be expected to contribute positively or negatively to the organism's success based on the success of the skeletal system. Evolution of bone shape must occur at the level of regulatory pathways, involving genes that do not directly specify bone shape. Evolution of bone mechanobiology, for example sensitivity and response to strain, may occur due to the organisms' success or failure while using the bone shapes that result.

Regional variation in allometry demonstrates the functional dependence of bone scaling. Future work will investigate gait and loading effects on bone allometry at multiple scale levels to continue the development of an integrative model of skeletal allometry.

## Materials and Methods

Cleaned felid appendicular bones (9 species, 13 specimens, 116 bones total) were obtained from the Natural History Museum, London, University Museum of Zoology Cambridge and post mortem. Body masses were not available for museum specimens. We did not calculate scaling exponents from body mass since true values were unknown for all but one specimen (*Felis catus*) and because felid body masses occupy broad intraspecific ranges (Table 5) [55]. Estimation of body mass is possible but requires back-calculation from bone dimensions [28], meaning that estimated body masses used in scaling comparisons would indirectly represent bone dimensions. To avoid this confounding situation we used only dimensions measured directly from CT scans for scaling calculations.

**Table 5:** Felid species.

Species	Common name	N	Body mass (kg)
*Felis catus*	Domestic cat	1	3–8
*Lynx canadensis*	Canadian lynx	1	5–17
*Leptailurus serval*	Serval	2	9–18
*Leopardus pardalis*	Ocelot	2	11–16
*Panthera pardus*	Leopard	1	28–90
*Acinonyx jubatus*	Cheetah	2	35–72
*Puma concolor*	Cougar	1	36–103
*Panthera tigris*	Tiger	2	100–306
*Panthera leo*	Lion	1	120–250

Felids used in the study are listed in order of minimum body mass [55].

*N*, number of specimens per species available for study.

Bones were selected from skeletally mature individuals showing no signs of degenerative joint disease, fracture or other disease. Bones containing active physes, osteophytosis, fracture callus or handling damage were excluded. Where possible, ipsilateral sets of scapula, humerus, radius, ulna, third metacarpal (Mc3), femur, tibia, fibula and third metatarsal (Mt3) were selected from at least one individual of each species. The fibula of *Panthera leo* was not available for CT scanning. Occasionally, bones from contralateral fore- and hindlimbs were selected due to missing or damaged bones from the ipsilateral sets; each full set contained bones from two limbs only, one forelimb and one hindlimb. Length, midshaft diameter and midshaft circumference were measured with dial callipers (±0.05 mm) and measuring tape (±0.5 mm). Diameters were measured in the craniocaudal and mediolateral directions. Scapular length was the distance between the distal and proximal extremes collinear with the scapular spine, and scapular width was the maximum width perpendicular to the scapular spine, regardless of position on the spine.

CT scans were made with a Picker PQ5000 (peak X-ray tube voltage 120 kVp; X-ray tube current 100 mA; exposure 64 mAs). Bones were supported on a radiolucent piece of elastomeric foam and aligned with their long axis parallel to the scanner's *z*-axis. The greatest practical resolution was used for each bone, with a maximum stack size of 200 slices and constant 512×512 pixels per slice. Small bones were scanned at higher resolution than large bones (pixel size 0.078–0.469 mm; slice thickness 1–2 mm). Digital oversampling was evident at high resolutions because pixel size was substantially smaller than true image resolution, which was measured as approximately 0.8 mm [56]. Images were exported to ImageJ (NIH, Maryland, USA) in 16-bit DICOM format with pixel values calibrated to Hounsfield units (HU). Imaging artefacts and extraneous anatomical features, such as articulating bones, were manually removed from images resulting in stacks containing contrast from only the bone of interest and air. Bone length was calculated by identifying the most proximal and distal bone points in the image stack, and the midslice was identified by taking the average slice number of the most proximal and most distal bone-containing slices. Bone length measured from CT stacks was strongly correlated with bone length measured directly (*R* ^2^ = 0.995). A total of 16435 slices of CT data were collected from the 13 specimens.

**Figure 5:**
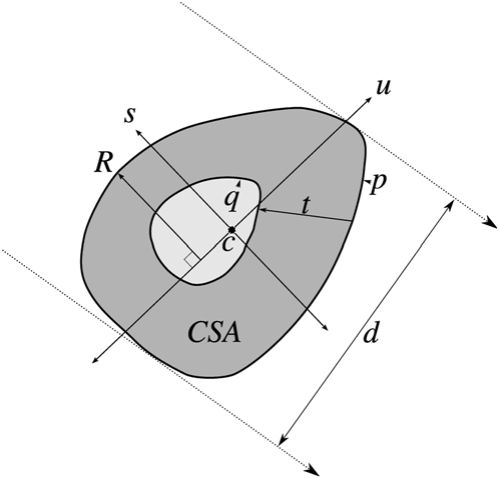
Slice measurements. The following calculations were made with an ImageJ macro for each CT slice after thresholding for cortical bone: centroid (*c*), major and minor principal axes (*u*, *s*) and the moments of inertia around them (*I*_min_, *I*_max_); outer and inner perimeters (*p*, *q*) were found; cortical thickness (*t*) was the distance from each point in *p* to the nearest point in *q*; cross-sectional area (*CSA*) was the number of thresholded pixels multiplied by pixel area; the greatest distance from each principal axis (*R*) was found for the calculation of *Z*_max_ and *Z*_min_; diameter (*d*) was the distance between two parallel lines of support as per the rotating calliper method. Equations are detailed in supplementary material (Text S2).

Image stacks were processed with an ImageJ macro (Text S1). Images were thresholded at 0 HU, which is the midpoint between air (−1000 HU) and cortical bone (1000 HU). Only pixels with values ≥0 HU contributed to geometric calculations. The macro calculated 11 parameters for each image slice: centroid (*x*_c_, *y*_c_); cross sectional area (*CSA*); minimum, maximum and mean cortical thickness (*t*_min_, *t*_max_, *t*_av_); maximum and minimum diameter (*d*_min_, *d*_max_); maximum and minimum second moment of area (*I*_max_, *I*_min_); and maximum and minimum section moduli (*Z*_max_, *Z*_min_) (Text S2). Second moments of area and section moduli were calculated directly from pixel coordinates without assumptions of cylindrical or elliptical geometry. Cortical thickness was calculated by wand selecting the inner and outer cortical boundaries, and for each point of the outer boundary finding the shortest distance to the inner boundary. Cortical thickness was not calculated for scapulae or fibulae, as these bones' cross-sectional geometries were irregular and lacked a consistent medullary cavity. Diameter was measured with the rotating callipers method [57] without assuming anatomic orientation (Figure 5).

The 3D centroid, principal axis eigenvectors and moments of inertia around the principal axes (*I*_1_, *I*_2_, *I*_3_) of each bone were determined with an ImageJ plugin (Text S3), assuming bone density of 1.8 g.cm^−3^ [58]. The plugin also rotated the CT data to align the bone image with its principal axes. To characterise joint geometry and its scaling, femoral head, humeral head and glenoid cavity radii were measured in ImageJ using a sphere-fitting technique previously validated for the humeral head and glenoid cavity of primates (Text S4) [59].

Data were collated into a MySQL (MySQL AB, Stockholm) database that was accessed directly with R (R Development Core Team, Vienna) for statistical analysis. Maximum section modulus (*Z*_max_) was normalised by dividing its cube root by bone length and plotted against per cent length, thus removing length from both axes so that proportional differences in *Z*_max_ between species would be apparent. Variables were log_10_ transformed prior to calculation of the regression slope. Allometric relationships were determined using the standardised major axis (SMA) method in the R package ‘smatr’ [60], [61] with the intercept not set. SMA was preferred over major axis (MA) for line-fitting due to SMA's greater precision and its ability to handle log transformed variables with arbitrary exponents [60]. Every slice's *z*-coordinate was labelled as per cent length and assigned to one of 20 bins, each of 5% length. Slice results were averaged within each bin and scaling exponents calculated for each bone type and bin.

Independence of data from correlation due to phylogeny was tested by constructing a phylogenetic tree for the selected felids (Figure S1) from which standardised contrasts were calculated (Text S5; Table S1). Scaling exponents of midslice dimensions against bone length were calculated with corrected regressions (Table S1).

## Supporting Information

Table S1 Midshaft allometry with phylogenetic correction. Tables detailing phylogenetic contrasts and all midshaft geometric parameters.
(0.15 MB XLS)

Text S1 ImageJ macro for the calculation of cross-sectional geometric parameters. This ImageJ macro sequentially analyses every slice of a CT stack, measuring several geometric parameters including second moment of area, section modulus and calliper diameter.
(0.03 MB TXT)

Text S2 Equation list. List of equations used to calculate cross-sectional and 3D geometric parameters.
(0.26 MB PDF)

Text S3 ImageJ plugin for calculation of 3D moments of inertia. This ImageJ plugin calculates 3D moments of inertia and rotates the image data into alignment with the 3D principal axes.
(0.01 MB TXT)

Text S4 ImageJ plugin for calculation of best-fit sphere. This ImageJ plugin takes a list of point selections from the ROI manager and returns the radius and centre of the best-fit sphere.
(0.00 MB TXT)

Text S5 Construction of the felid cladogram for phylogenetic contrasts. Justification for the construction of the felid cladogram and description of the phylogenetic control applied to the allometric calculations.
(0.11 MB PDF)

Figure S1 Felid cladogram. Phylogenetic relationship of felids used to calculate allometric relationships. Numbers indicate branch length in millions of years.
(0.31 MB PNG)
