# Correction: Three-Dimensional Geometric Analysis of Felid Limb Bone Allometry

**DOI:** 10.1371/annotation/5435c6bd-12d3-4bb4-97b7-4e96d4773525

**Published:** 2009-04-06

**Authors:** Michael Doube, Alexis Wiktorowicz-Conroy, Per Christiansen, John R. Hutchinson, Sandra Shefelbine

The second author's name is displayed incorrectly in the by-line and the citation. The name should read: Alexis Wiktorowicz-Conroy. The citation should read: Doube M, Wiktorowicz-Conroy A, Christiansen P, Hutchinson JR, Shefelbine S (2009) Three-Dimensional Geometric Analysis of Felid Limb Bone Allometry. PLoS ONE 4(3): e4742. doi:10.1371/journal.pone.0004742

