# Supplementary material for: Three-Dimensional Geometric Analysis of Felid Limb Bone Allometry
Source: PLoS One. 2009 Mar 9;4(3):e4742. doi: 10.1371/journal.pone.0004742 (PMC2650414; doi:10.1371/journal.pone.0004742)
Supplement: Text S2 — Equation list. List of equations used to calculate cross-sectional and 3D geometric parameters. (0.26 MB PDF) [file pone.0004742.s003.pdf]

Three-Dimensional Geometric Analysis in Felid Limb-Bone Allometry: Equation list.

Number of thresholded pixels (n), pixel width (w), height (h) and voxel depth (d) are used throughout. All coordinates are given in mm.

Centroid (x_c, y_c)

$$(x_c, y_c)=\left(\sum_{i=1}^{n} x , \sum_{i=1}^{n} y\right)$$

Second moments of area relative to x and y axes

$$I_y=\sum_{i=1}^{n}\left(w\cdot h\cdot(x-x_c)^2+\frac{w^3\cdot h}{12}\right)$$

$$I_x=\sum_{i=1}^{n}\left(w\cdot h\cdot(y-y_c)^2+\frac{h^3\cdot w}{12}\right)$$

$$I_{xy}=\sum_{i=1}^{n}\left(w\cdot h\cdot(x-x_c)\cdot(y-y_c)\right)$$

Angle of principal axis (radians)

$$\theta=\arctan\left(\frac{I_x-I_y+\sqrt{(I_x-I_y)^2+4\cdot I_{xy}^2}}{2\cdot I_{xy}}\right)$$

Maximum ($I_{\max}$) and minimum ($I_{\min}$) second moments of area

$$I_{max}=\frac{I_x+I_y}{2}+\sqrt{\frac{(I_x-I_y)^2}{2}+I_{xy}^2}$$

$$I_{min}=\frac{I_x+I_y}{2}-\sqrt{\frac{(I_x-I_y)^2}{2}+I_{xy}^2}$$

Polar moment of inertia

$$J_z=I_{max}+I_{min}$$

Maximum distance of a thresholded pixel from principal axes

(x_c, y_c) = centroid
R_{max} = maximum distance from maximum principal axis
R_{min} = maximum distance from minimum principal axis

For all thresholded pixels (x, y):

$$R_{max} = max\{|(x-x_c)\cdot\cos\theta + (y-y_c)\cdot\sin\theta|\}$$

$$R_{min} = max\{|(y-y_c)\cdot\cos\theta - (x-x_c)\cdot\sin\theta|\}$$

Section moduli

$$Z_{max} = \frac{I_{max}}{R_{max}}$$

$$Z_{min} = \frac{I_{min}}{R_{min}}$$

3D centroid

$$(x_c, y_c, z_c) = \left(\sum_{i=1}^{n} x, \sum_{i=1}^{n} y, \sum_{i=1}^{n} z\right)$$

Inertia Tensor

$$M = \begin{bmatrix} I_{xx} & I_{xy} & I_{xz} \\ I_{yx} & I_{yy} & I_{yz} \\ I_{zx} & I_{zy} & I_{zz} \end{bmatrix}$$

Moments of inertia
(x_c, y_c, z_c) is the 3D centroid
ρ is density of whole object
V is volume of whole object

$$I_{xx} = \sum_{i=1}^{n} \left((y-y_c)^2 + (z-z_c)^2 + \frac{h^2+d^2}{12}\right)\cdot\rho\cdot V$$
$$I_{yy} = \sum_{i=1}^{n} \left((x-x_c)^2 + (z-z_c)^2 + \frac{w^2+d^2}{12}\right)\cdot\rho\cdot V$$
$$I_{zz} = \sum_{i=1}^{n} \left((y-y_c)^2 + (x-x_c)^2 + \frac{h^2+w^2}{12}\right)\cdot\rho\cdot V$$

$$I_{xy} = -\sum_{i=1}^{n} \left((x-x_c)\cdot(y-y_c)\right)\cdot\rho\cdot V$$
$$I_{xz} = -\sum_{i=1}^{n} \left((x-x_c)\cdot(z-z_c)\right)\cdot\rho\cdot V$$
$$I_{zy} = -\sum_{i=1}^{n} \left((z-z_c)\cdot(y-y_c)\right)\cdot\rho\cdot V$$
