# Supplementary material for: Three-Dimensional Geometric Analysis of Felid Limb Bone Allometry
Source: PLoS One. 2009 Mar 9;4(3):e4742. doi: 10.1371/journal.pone.0004742 (PMC2650414; doi:10.1371/journal.pone.0004742)
Supplement: Text S5 — Construction of the felid cladogram for phylogenetic contrasts. Justification for the construction of the felid cladogram and description of the phylogenetic control applied to the allometric calculations. (0.11 MB PDF) [file pone.0004742.s006.pdf]

Construction of Felid Phylogeny for Corrected Regressions

Owing to the error terms (ε) in traditional regression analyses on biological data invariably being correlated, as indicated by the branching structure of phylogenetic relationships, implying that individual species data are not truly independent [1-3], we also employed independent contrasts regression analysis [4-7]. We assembled a phylogenetic tree for the included species of felids based on literature sources, with estimated divergence times based on fossil evidence and tree topology.

The age of the Pantherinae clade has been controversial. Based on 12S RNA and cytochrome b sequences, Janczewski et al. (1995) concluded that the Pantherinae was the youngest of the extant felid lineages [8]; in contrast, based on 16S rRNA and NADH-5 genes [9], and nuclear genes [10], it was concluded that it was the oldest. There is no fossil evidence to support a Miocene age of the Pantherinae, as inferred by such studies; the oldest fossils provisionally to *Panthera* sp. are slightly older than 3.5 MYA [11-14]. All hitherto known fossils of great cats are Plio-Pleistocene, and fossil evidence from the entire world indicate that members of this clade only became numerous and began differentiating in the Late Pliocene-Pleistocene.

Isolated fossils of purported leopards (*Panthera pardus*) are known from ~3.8 MYA deposits in Africa [11-13,15], but their identity has yet to be conclusively established. They may instead belong to a more stem-based taxon within *Panthera*, or may, in fact, represent primitive pumas [16,17], or even cheetahs [18]; molecular studies indicate that modern lineages of the leopard did not begin to differentiate until within the last ~1 MYA [19,20]. Fossils from Laetoli, Tanzania, dated at >3.5 MYA have been attributed to lions (*Panthera leo*) [12-15], but this is uncertain [11], and a more likely interpretation is that they belong to a more primitive, stem-taxon within *Panthera* (Hemmer et al. 2001) [16]. Lion-like cats attributable to the Eurasian cave lion (*Panthera fossilis/spelaea*) are much younger, and are known from numerous Eurasian sites from the Early-Late Pleistocene [21-27]. The age of *Panthera onca*/*gombaszoegensis* fossils is also Late Pliocene-Pleistocene [16,28,29]; the jaguar lineage is the sister-taxon of the lion+leopard lineage [16,30]. Accordingly, we set the age of the lion and leopard lineages at 2 MYA, and added another 0.5 MYA to encompass the age of the lion+leopard and their immediate relatives before joining with the more primitive tiger.

The modern tiger (*Panthera tigris*) is probably around 1.5 MYA; the lineage to which it belongs was set at 2.5 MYA, to accommodate the inferred age of the lion+leopard lineage. Tiger fossils are known from the Lower Pleistocene of Central and South-east Asia [31-35], and the differentiation of the tiger probably occurred in the early Pleistocene [36]. The so-called "paleo-tiger" *Panthera palaeosinensis* is known from the mid-Pleistocene [37], and although it has been referred to as a primitive tiger [38], it represents a more ancestral species of *Panthera* [30]. Thus, although new finds are Late Pliocene [18], making it one of the oldest well-known pantherine species, this has no direct bearing on the inferred age of the tiger lineage.

Pumas and cheetahs are closely related [8,10,39]. Fossil remains of the puma (*Puma concolor*) are only known from the Rancholabrean (Late Pleistocene; ~400 KYA) [28,39-41]. The fossil puma-like cat *Puma pardoides* is known from the early-middle Villafranchian (Late Pliocene) [42,43], and isolated finds from Kvabebi are older than 2.6 MYA [17]. "*Puma*" *lacustris* from the Blancan may or may not be a primitive type of puma [44]. The oldest cheetahs (*Acinonyx* sp.) are also Late Pliocene (~2.5 MYA) [18,45,46]. Accordingly, we inferred the ages of the *Puma* and *Acinonyx* lineages as 3 MYA each; molecular studies have previously inferred an age for the *Puma* lineage of 3.2 MYA [47]. The closely related fossil clade of *Miracinonyx* is also no older than ~3.2 MYA [39], suggesting that an overall age of the entire clade of perhaps 4-5 MYA. Thus, another 1.5 MYA was added to give the *Puma-Acinonyx* clade an overall age of 4.5 MYA, slightly less than the ~5 MYA suggested by Janczewski et al. (1995), and Johnson et al. (2006), and far less than >8 MYA suggested by Johnson & O´Brien (1997) [8-10].

The domestic cat (*Felis catus*) is one of the last animals to become domesticated by man, and although the domestication is believed to have occurred around 4000 years ago in Egypt [48], fragmentary finds indicate an age of perhaps 6000 years [49]. The domestic cat originated from the North-African wildcat (*Felis silvestris libyca*), and their great osteological resemblance makes older

(6-7000 years), archaeological finds of purported domestic cats tentative, because these may have been from wildcats killed for their fur [50]. We infer a total age for this clade of 4.5 MYA to accommodate the lineage of *Puma+Acinonyx* and their close relatives.

The oldest well-known member of the *Lynx* lineage is *Lynx issiodorensis* from the Blancan of North America [28,44,51], but fragmentary finds indicate a possible Late Hemphillian (Late Miocene–Early Pliocene; >5MYA) for the *Lynx* lineage [44,52]. To accommodate this, we assigned an age of 5.5 MYA to this lineage.

The fossil record of the ocelot (*Leopardus pardalis*) is scarce, and includes several fragmentary finds from the North American Pleistocene [53,54]; most are Irvingtonian, but the species may well have persisted into the Sangamonian [55]; it is still found in the southern USA today [56,57]. The lineage was inferred to be 5.1 MYA by Mattern & McLennan (2000), but only 2.9 MYA by Johnson et al. (2006) [10,58]. We infer an age of 6.5 MYA for this entire lineage, to accommodate the age of *Lynx-Acinonyx*.

Fossil material referred to as *Leptailurus* sp., and thus on the lineage of the extant serval (*Leptailurus serval*) has been recovered at Laetoli in Tanzania and Lothagam in Kenya in deposits with an estimated age of 3.8-3.5 MYA, and 3-2.5 MYA, respectively [15]. We infer an age of 7.5 MYA for this entire lineage, to accommodate the age of the sister-clade of *Leopardus-Acinonyx*.

Independent contrasts for the included 3D variables were computed by subtraction of values in two sister taxa, or in a given taxon from the nearest tree node, as appropriate. Prior to regression analysis, the contrasts have to be standardized, which implies bringing them to a common variance, which was done by dividing each individual contrast by the square root of the sum of all the branch lengths that make up the contrast in question. Proper standardization implies that the resulting common variance be independent of the branch lengths, and this may be analysed by application of several methods, of which examination of plots of standard deviations of contrasts to absolute values of contrasts appear to be the most reliable and best understood [5-7,59,60]. We analysed such plots by visual inspection and by computing correlation coefficients between the standard deviations of the contrasts and the absolute values of the contrasts. Initially, the unmodified branch lengths (in MYA) were examined, and if these were found to show any discernible structure and have a significant ($p \geq 0.05$) correlation coefficient, we transformed the branch lengths by taking the square root ($\sqrt{}$); the cube root ($\sqrt[3]{}$), the natural logarithm ($\log_{10}$); and Grafens arbitrary branch length [1], respectively, and ran the analysis again. The approach which resulted in the lowest correlation coefficient was applied in the subsequent analysis of independent contrasts.

Independent contrasts analysis produces a regression through the origin and, accordingly, no intercept. We used $\log_{10}$ bone lengths as the independent variable, and the various $\log_{10}$ 3D data as dependent variables. However, owing to bone length not constituting a truly independent variable in a mathematical sense, as error must be assumed on both variables even using 3D scanning data, and since we feel it is unwarranted to assign statistical dependence to any one variable *a priori*, we decided to employ the Reduced Major Axis (model II) regression approach, since this assumes no dependence of one variable on another. We conducted independent contrast analysis in the program PDTREE [6,7,61,62], which only reports 95% confidence intervals for Least Squares (Model I) regression slope; however, as can be done for traditional regression analysis, we used the standard errors of the Least Squares regression analysis to estimate the confidence interval for the slope in Reduced Major Axis analysis.

References

1. Grafen A (1989) The Phylogenetic Regression. Phil Trans R Soc Lond B 326: 119-157. doi:10.1098/rstb.1989.0106

2. Grafen A (1992) The uniqueness of the phylogenetic regression. J Theor Biol 156: 405-423.

3. Harvey PH, Pagel MD (1991) The Comparative Method in Evolutionary Biology. Oxford: Oxford University Press. 239 p.

4. Felsenstein J (1985) Phylogenies and the Comparative Method. Am Nat 125: 1-15.

5. Garland T, Harvey PH, Ives AR (1992) Procedures for the Analysis of Comparative Data Using Phylogenetically Independent Contrasts. Syst Biol 41: 18-32.

6. Garland T, Midford PE, Ives AR (1999) An Introduction to Phylogenetically Based Statistical Methods, with a New Method for Confidence Intervals on Ancestral Values. Am Zool 39: 374-388. doi:10.1093/icb/39.2.374

7. Garland T, Ives AR (2000) Using the past to Predict the Present: Confidence Intervals for Regression Equations in Phylogenetic Comparative Methods. Am Nat 155: 346-364.

8. Janczewski DN, Modi WS, Stephens JC, O'Brien SJ (1995) Molecular evolution of mitochondrial 12S RNA and cytochrome b sequences in the pantherine lineage of Felidae. Mol Biol Evol 12: 690-707.

9. Johnson WE, O'Brien SJ (1997) Phylogenetic Reconstruction of the Felidae Using 16S rRNA and NADH-5 Mitochondrial Genes. J Mol Evol 44: S098-S116. doi:10.1007/PL00000060

10. Johnson WE, Eizirik E, Pecon-Slattery J, Murphy WJ, Antunes A, et al. (2006) The late Miocene radiation of modern Felidae: a genetic assessment. Science 311: 73-7. doi:311/5757/73

11. Barry JC (1987) Large carnivores (Canidae, Hyaenidae, Felidae) from Laetoli. In: Leakey MD, Harris JM, editors. Laetoli - a Pliocene site in Tanzania. Oxford: Clarendon Press. pp. 235-258.

12. Turner A (1990) The evolution of the guild of larger terrestrial carnivores during the Plio-Pleistocene in Africa. Geobios 23: 349-368. doi:10.1016/0016-6995(90)80006-2

13. Turner A (1990) Late Neogene/Lower Pleistocene Felidae of Africa: evolution and dispersal. Quartärpaläontologie 8: 247-256.

14. Turner A, Antón M (1997) The Big Cats and Their Fossil Relatives: An Illustrated Guide to Their Evolution and Natural History. New York: Columbia University Press. 234 p.

15. Werdelin L, Lewis ME (2005) Plio-Pleistocene Carnivora of eastern Africa: species richness and turnover patterns. Zool J Linn Soc 144: 121-144. doi:10.1111/j.1096-3642.2005.00165.x

16. Hemmer H, Kahlke R, Vekua A (2001) The Jaguar - *Panthera onca gombaszoegensis* (Kretzoi, 1938) (Carnivora: Felidae) in the late lower pleistocene of Akhalkalaki (south Georgia; Transcaucasia) and its evolutionary and ecological significance. Geobios 34: 475-486. doi:10.1016/S0016-6995(01)80011-5.

17. Hemmer H, Kahlke R, Vekua A (2004) The Old World puma – *Puma pardoides* (Owen, 1846) (Carnivora: Felidae) – in the Lower Villafranchian (Upper Pliocene) of Kvabebi (East Georgia, Transcaucasia) and its evolutionary and biogeographical significance. N Jahrb Geol Paläontol Abh 233: 197-231.

18. Christiansen P, Mazák J (2008) A primitive Late Pliocene cheetah and evolution of the cheetah lineage. Proc Natl Acad Sci U S A: in press.

19. Miththapala S, Seidensticker J, O'Brien SJ (1996) Phylogeographic Subspecies Recognition in

Leopards (*Panthera pardus*): Molecular Genetic Variation. Cons Biol 10: 1115-1132.

20. Uphyrkina O, Johnson WE, Quigley H, Miquelle D, Marker L, et al. (2001) Phylogenetics, genome diversity and origin of modern leopard, *Panthera pardus*. Mol Ecol 10: 2617-2633. doi:10.1046/j.0962-1083.2001.01350.x

21. Hilzheimer M (1924) Die systematische Stellung von *Felis spelaea* Goldf. Sitzber Ges Naturforsch Freund 1922: 11-24.

22. Hilzheimer M (1940) Contribution á l'étude de *Felis spelaea* Goldf. Mel Préhist d'Anthropol 1939: 43-50.

23. Koby FE (1941) Contribution á l'étude de *Felis spelaea* Goldf. Verh Naturforsch Ges Basel 52: 168-188.

24. Kabitzsch J- (1960) Die Verwandtschaft vom Löwen und Tiger dargestellt in ihrem Gebiss unter Berücksichtigung der Gebisse von Jaguar und den zwei pleistozänen Großkatzen *Felis spelaea* und *Felis arox*. Säugetierk Mitt 8: 103-140.

25. Dietrich WD (1968) Fossile Löwen im europäischen und afrikanischen Pleistozän. Paläontol Abh (Abt A) 3: 323-366.

26. Kurtén B (1968) Pleistocene mammals of Europe. London: Weidenfield & Nicholson. 317 p.

27. Hemmer H (1974) Untersuchungen zur Stammesgeschichte der Pantherkatzen (Pantherinae). Teil III. Zur Artsgeschichte des Löwen *Panthera* (*Panthera*) *leo* (Linnaeus 1758). Veröff Zool Staatssamlung München 17: 167-280.

28. Kurtén B, Anderson E (1980) Pleistocene Mammals of North America. New York: Columbia University Press. 442 p.

29. O'Regan HJ (2003) A phylogenetic and palaeoecological review of the Pleistocene felid *Panthera gombaszoegensis*. Quatern Newsl 99: 81.

30. Christiansen P (2008) Phylogeny of the great cats (Felidae: Pantherinae), and the influence of fossil taxa and missing characters. Cladistics. doi:10.1111/j.1096-0031.2008.00226.x

31. Zdansky O (1928) Die Säugetiere der Quartärfauna von Chou-Kóu-Tien. Palaeontol Sinica 5: 1-146.

32. Brongersma LD (1935) Notes on some recent and fossil cats chiefly from the Malay Peninsula. Zoologische Mededeelingen 18: 1-89.

33. Teilhard de Chardin P, Young CC (1936) On the mammalian remains from the archaeological site of Anyang. Palaeontol Sinica (Ser C) 12: 1-61.

34. Hooijer DA (1947) Pleistocene remains of *Panthera tigris* (Linnaeus) subspecies from Wanhsien, Szechwan, China, compared with fossil and Recent tigers from other localities. Am Mus Nov 1346. Available: http://hdl.handle.net/2246/2316. Accessed 22 Oct 2008.

35. Tscherski JD (1892) Beschreibung der Sammlung posttertiärer Säugethiere. Mém Acad Imp St Pétersbourg (Ser 7) 40: 1-511.

36. Mazák V (1979) Der Tiger. 2nd ed. Wittenberg Lutherstadt: Ziemsen. 228 p.

37. Pei W- (1934) On the Carnivora from locality 1 of Choukoutien. Palaeontol Sinica 8: 1-216.

38. Hemmer H (1967) Wohin gehört „*Felis*“ *palaeosinensis* Zdansky, 1924, in systematischer Hinsicht? N Jahrb Geol Paläontol Abh 129: 83-96.

39. Van Valkenburgh B, Grady F, Kurtén B (1990) The Plio-Pleistocene cheetah-like cat *Miracinonyx inexpectatus* of North America. J Vert Paleontol 10: 434-454.

40. Kurtén B (1976) Fossil puma (Mammalia: Felidae) in North America. Netherlands J Zool 26: 502-534.

41. Morgan GS, Seymour KL (1997) Fossil history of the panther (*Puma concolor*) and the cheetah-like cat (*Miracinonyx inexpectatus*) in Florida. Bull Florida Mus Nat Hist 40: 177-219.

42. Viret J (1954) Le Loess a bancs Durcis de Saint-Vallier (Drome) et sa Faune de Mammiféres Villafranchiens. Nouv Arch Mus D’Hist Nat Lyon 4: 1-200.

43. Kurtén B, Crusafont-Pairó M (1977) Villafranchian carnivores (Mammalia) from La Puebla de Valverde (Teruel, Spain). Comment Biol Soc Sci Fenn 85: 1-39.

44. Glass GE, Martin LD (1978) Multivariate comparison of some extant and fossil felidae. Carnivore 1: 80-87.

45. Sotnikova MV (1978) Upper Pliocene Carnivora of Central Asia. Intl Geol Rev 20: 335-338.

46. Geraads D (1997) Carnivores du Pliocène terminalde Ahl al Oughlam (Casablanca, Maroc). Geobios 30: 127-164. doi:10.1016/S0016-6995(97)80263-X

47. Barnett R, Barnes I, Phillips MJ, Martin LD, Harington CR, et al. (2005) Evolution of the extinct Sabretooths and the American cheetah-like cat. Curr Biol 15: R589-R590. doi:10.1016/j.cub.2005.07.052

48. Clutton-Brock J (1987) A natural history of domesticated animals. Cambridge University Press.

49. Davis SJM (1987) The Archaeology of Animals. London: Batsford. 208 p.

50. Clutton-Brock J (1979) The mammalian remains from the Jericho Tell. Proc Prehist Soc 45: 135-158.

51. Kurtén B (1963) Return of a lost structure in the evloution of the felid dentition. Soc Scient Fenn Comment Biol 26: 3-11.

52. MacFadden B, Galiano H (1981) Late Hemphillian cat (Mammalia, Felidae) from the Bone Valley Formation of Central Florida. J Paleontol 55: 218-226.

53. Ray CE, Olson SJ, Gut HJ (1963) Three mammals new to the Pleistocene fauna of Florida, and a reconsideration of five earlier records. J Mammol 44: 373-395.

54. Kurtén B (1965) The Pleistocene Felidae of Florida. Bull Florida State Mus 9: 215-273.

55. Werdelin L (1985) Small Pleistocene Felines of North America. J Vert Paleontol 5: 194-210.

56. Murray JL, Gardner GL (1997) *Leopardus pardalis*. Mamm Spec 548: 1-10.

57. Sunquist M, Sunquist F (2002) Wild Cats of the World. Chicago: University of Chicago Press. 452 p.

58. Mattern MY, McLennan DA (2000) Phylogeny and Speciation of Felids. Cladistics 16: 232-253. doi:10.1111/j.1096-0031.2000.tb00354.x

59. Garland T (1992) Rate Tests for Phenotypic Evolution Using Phylogenetically Independent Contrasts. Am Nat 140: 509-519.

60. Díaz-Uriarte R, Garland TJ (1996) Testing hypotheses of correlated evolution using phylogenetically independent contrasts: sensitivity to deviations from Brownian motion. Syst Biol 45: 27-47.

61. Garland T, Dickerman AW, Janis CM, Jason A. Jones (1993) Phylogenetic Analysis of Covariance by Computer Simulation. Syst Biol 42: 265-292.

62. Garland T, Midford PE, Jones JA, Dickerman AW, Díaz-Uriarte R. PDAP: Phenotypic Diversity Analysis Programs.
