# Supplementary figures and images for: Three-Dimensional Geometric Analysis of Felid Limb Bone Allometry
Source: PLoS One. 2009 Mar 9;4(3):e4742. doi: 10.1371/journal.pone.0004742 (PMC2650414; doi:10.1371/journal.pone.0004742)

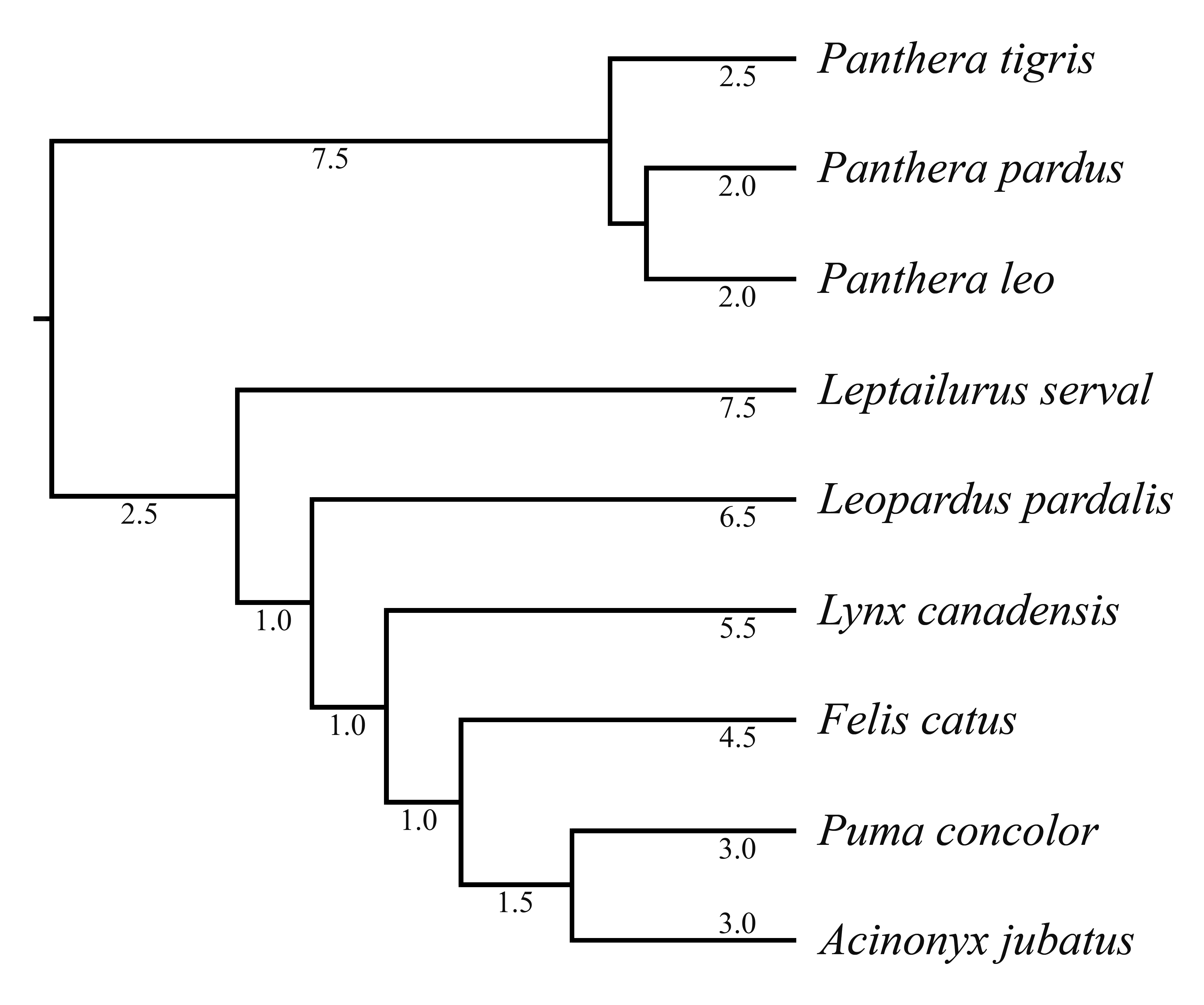

Supplement: Figure S1 — Felid cladogram. Phylogenetic relationship of felids used to calculate allometric relationships. Numbers indicate branch length in millions of years. (0.31 MB PNG) [file pone.0004742.s007.png]
